# Biologically Active Oxylipins from Enzymatic and Nonenzymatic Routes in Macroalgae

**DOI:** 10.3390/md14010023

**Published:** 2016-01-20

**Authors:** Mariana Barbosa, Patrícia Valentão, Paula B. Andrade

**Affiliations:** REQUIMTE/LAQV, Laboratory of Pharmacognosy, Department of Chemistry, Faculty of Pharmacy, University of Porto, Rua de Jorge Viterbo Ferreira No. 228, Porto 4050-313, Portugal; mariana.nunes.barbosa@gmail.com (M.B.); valentao@ff.up.pt (P.V.)

**Keywords:** oxylipins, macroalgae, phytoprostanes, biosynthesis, bioactivity

## Abstract

Marine algae are rich and heterogeneous sources of great chemical diversity, among which oxylipins are a well-recognized class of natural products. Algal oxylipins comprise an assortment of oxygenated, halogenated, and unsaturated functional groups and also several carbocycles, varying in ring size and position in lipid chain. Besides the discovery of structurally diverse oxylipins in macroalgae, research has recently deciphered the role of some of these metabolites in the defense and innate immunity of photosynthetic marine organisms. This review is an attempt to comprehensively cover the available literature on the chemistry, biosynthesis, ecology, and potential bioactivity of oxylipins from marine macroalgae. For a better understanding, enzymatic and nonenzymatic routes were separated; however, both processes often occur concomitantly and may influence each other, even producing structurally related molecules.

## 1. Introduction

Fatty acids are key components of cell membranes and storage lipids in all living organisms. These central building blocks are prone to undergoing oxidation reactions through both enzymatic and nonenzymatic cellular mechanisms. The biosynthesis of oxygenated derivatives of polyunsaturated fatty acids (PUFA), collectively termed oxylipins, is highly dynamic and occurs as both a developmentally regulated mode and a response to abiotic and biotic stresses. The oxylipin pathway is initiated by the formation of fatty acid hydroperoxydes, either by chemical (auto)oxidation induced by free radicals and reactive oxygen species (ROS), or catalyzed by enzymes, such as lipoxygenases (LOX) [1]. The primary hydoperoxyde products are further converted into a large variety of oxylipin classes, through an array of alternative and subsequent reactions, having crucial signaling functions in different organisms [2,3,4,5,6,7,8,9,10]. In fact, oxylipins’ cellular functions are as diverse as oxylipins themselves [1]. This family of structurally diverse metabolites is ubiquitously distributed in nature, being found in animals, plants, bacteria, mosses, and algae [2,11].

Due to the wealth of novel oxylipin structures encountered in marine organisms, the uniqueness of their biosynthetic pathways, and the potency of their biological effects, marine oxylipins have been recent targets of lipid research. In fact, over the last decades researchers have focused their attention on the isolation, structural elucidation, and biological properties of oxylipins from marine organisms, which have emerged as incredibly rich sources of these low-molecular-weight lipids. The overwhelming majority of marine oxylipins derive from LOX metabolism of PUFA precursors with a variety of carbon lengths (C_16_ to C_22_) and unsaturation patterns (ω3, ω6, ω9) [3].

## 2. Oxylipin Biosynthesis in Macroalgae

**Figure 1:**
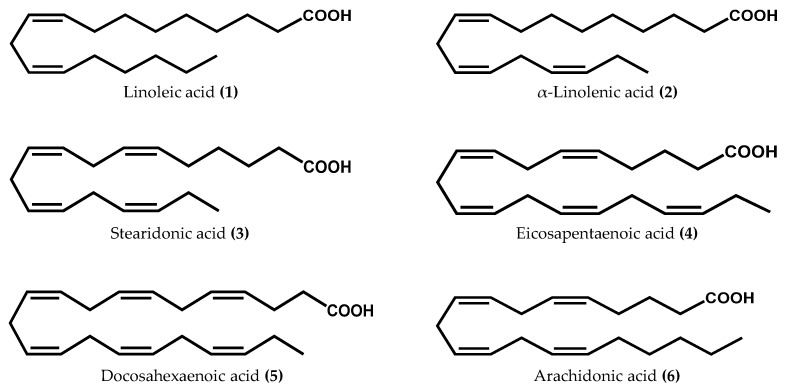
Chemical structures of the main fatty acids used as substrates in the biosynthesis of oxylipins in macroalgae.

Macroalgae comprise an abundant and heterogeneous group of marine organisms characterized by their photosynthetic nature and worldwide distribution [12]. Biodiversity within red (Rhodophyta), green (Chlorophyta), and brown (Ochrophyta) macroalgae offers the possibility of finding a wide variety of natural compounds with interesting properties [13]. Among the great chemical diversity, macroalgae are unanimously acknowledged as the main primary producers of PUFA. Some major algal PUFA, including the human-essential linoleic (**1**) and α-linolenic (**2**) acids, as well as stearidonic (**3**), eicosapentaenoic (**4**), and docosahexaenoic (**5**) acids (Figure 1), are not only important membrane components, but may also be involved in the regulation of physiological processes, by serving as precursors in the biosynthesis of a multitude of structurally unique oxylipins [14,15,16,17,18,19,20,21,22,23,24]. Surprisingly, in several cases the abundance of PUFA substrates does not closely parallel their use in oxylipin biosynthetic pathways. For instance, macroalgae belonging to the Ochrophyta phylum are relatively depauperate in C_18_ PUFA; however, they commonly utilize this substrate in LOX-initiated biosynthetic pathways. Chlorophyta, like higher terrestrial plants, mainly oxidizes C_18_ substrates, while Rhodophyta seems to fully utilize C_18_ and C_20_ PUFA for oxylipin generation [3].

Nevertheless, and based on the algal oxylipin structures identified so far, it has been generally accepted that both eicosanoid and octadecanoid pathways can be found in these photosynthetic marine organisms [25]. However, the information available on algal oxylipin biosynthesis is still very scarce and most of the data originate from metabolic studies.

Despite the discovery of diverse oxylipins in all of the algal phyla, only recently have researchers deciphered the functional roles of some of these metabolites in the defense and innate immunity of macroalgae [26,27,28,29,30,31,32,33,34]. Macroalgae have no acquired immune system, strongly depending on their chemical repertoire to mediate interactions with other organisms and with the environment [35]. They resemble terrestrial plants and animals in their basic mechanisms for pathogen recognition and defense signaling [36]. In plants, oxylipins play a pivotal role in host–microbe interactions, stimulate the expression of genes encoding proteins involved in the defense against pathogens, and regulate growth and development [4,7,37]. In mammals oxylipins, such as leukotrienes and prostaglandins, are known to operate in inflammatory processes, allergic responses, and, in a broader sense, defensive stress responses to infection, drugs, and xenobiotics [38]. Likewise, in algal systems oxidized fatty acid derivatives appear to be involved in systemic defense mechanisms, accumulating in response to wounds [27,32], pathogen infection [26], metal toxicity [39,40,41,42], desiccation [43,44], and other kinds of stress [45,46,47].

### 2.1. Enzymatically-Derived Algal Oxylipins

**Figure 2:**
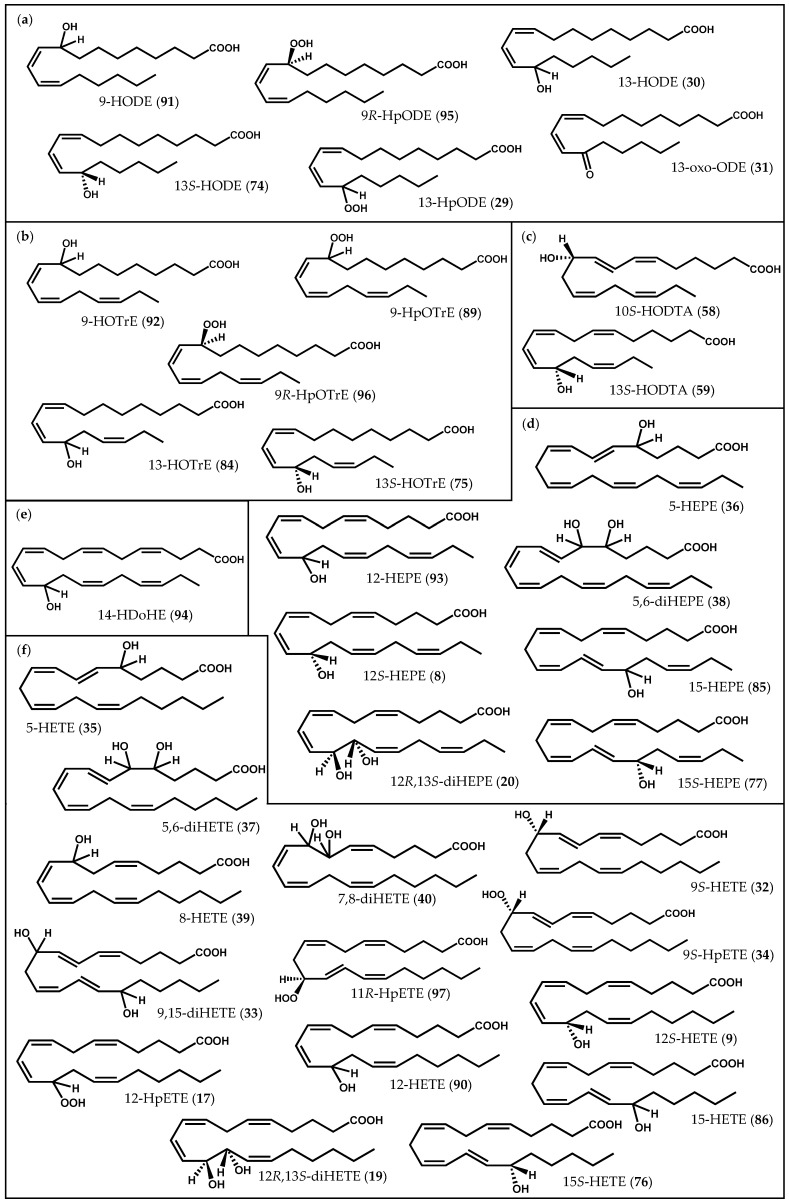
Chemical structures of some common oxylipins described in macroalgae: (**a**) linoleic acid derivatives; (**b**) α-linolenic acid derivatives; (**c**) stearidonic acid derivatives; (**d**) eicosapentaenoic acid derivatives; (**e**) docosahexaenoic acid derivatives; (**f**) arachidonic acid derivatives.

Enzymatically-derived oxylipin biosynthesis is a multi-step process commonly initiated by LOX, a large family of non-heme iron-containing enzymes that catalyzes the stereo- and regio-specific insertion of molecular oxygen into PUFA substrates containing a (1*Z*,4*Z*)-pentadiene system [48]. In higher plants, for instance, the catalytic insertion of oxygen either takes place at C-9 or C-13 of a C_18_ PUFA hydrocarbon backbone, resulting in the formation of two groups of compounds: 9*S*-hydroperoxy and 13*S*-hydroperoxy-derived oxylipins. In mammals, however, the prototypical substrate (arachidonic acid (**6**), Figure 1) can be oxygenated by LOX at six different positions (C-5, C-8, C-9, C-11, C-12, or C-15) [49]. Regarding macroalgae, studies have suggested that the octadecanoid metabolic pathway may be inherited from the chloroplast and that the eicosanoid pathway is probably inherited from the ancient eukaryotes [36]. Therefore, macroalgae are capable of metabolizing C_18_ PUFA at C-9, C-11, and C-13 via 9-, 11-, and 13-LOX, respectively, while C_20_ PUFA are transformed at C-5, C-8, C-9, C-12, and C-15 via 5-, 8-, 12-, and 15-LOX, respectively [2]. Similarly to higher terrestrial plants and mammals, the resulting hydroperoxides are further converted to a broad range of oxygenated derivatives, such as hydroxy-, oxo-, epoxy- fatty acids, and polyunsaturated aldehydes (PUA), by the activity of LOX, peroxygenases, oxygenases, epoxygenases, and hydroperoxide lyases (HPL), respectively [17,26,40,50]. Some red algae also form prostaglandins and leukotrienes, either nonenzymatically or by the enzymatic action of allele oxide synthase/cyclase (AOS/AOC) or cycloxygenase (COX) analogous to animals [2,51]. Apart from common oxygenated fatty acid derivatives (Figure 2), macroalgae also contain a number of complex and unique oxylipins, such as cyclopropyl hydroxyeicosanoids, egregiachlorides, ecklonialactones, hybridalactones, bicyclic cymathere ethers, cymatherelactones, and cymatherols [18,22,23,52].

Although extensive detail about oxylipin occurrence in microalgae is beyond this review, some important features of oxylipin metabolism in these unicellular organisms cannot be discarded. A characteristic difference from macroalgae is the complete absence of C_18_ PUFA-derived LOX products in several species of diatom microalgae [2]. Diatoms, which have emerged as an independent lineage quite recently in the evolution of photosynthetic eukaryotes, use eicosapentaenoic acid (**4**) and chloroplastic C_16_ fatty acids as substrates for oxylipin assembly [53]. Furthermore, only a few additional accounts of oxylipins from other microalgae classes have been addressed, most of which are restricted to common hydroxy-fatty acid derivatives [54,55,56,57,58,59,60].

The great diversity of oxylipins in macroalgae is partly explained by the differences in the oxygenation position, mainly catalyzed by LOX, and the variability of the downstream reactions. Nevertheless, a very limited number of genome sequences and, therefore, of enzymes related to oxylipin pathways, is yet available, making the source of these abundant oxylipins in algae a puzzle.

A cDNA clone encoding a putative 12-LOX (Ppu LOX) was identified, for the first time, in the gametophytes of *Porphyra purpurea* (Roth) C. Agardh by Liu & Reith [61]. This sequence showed low percent identity (25%–30%) to both mammalian and plant LOX, establishing a separate phylogenetic branch from the other known LOX sequences [61].

Zhu *et al.* [49] and Chen *et al.* [62] have now disclosed the full sequences of two LOX genes (PhLOX and PhLOX2) from the gametophytes of the red algae *Pyropia haitanensis* (T.J. Chang & B.F. Zheng) N. Kikuchi & M. Miyata. Similarly to Ppu LOX, PhLOX2 presented a low percent identity (<31%) to the mammalian and plant LOX and exhibited remarkable substrate and position flexibility, being able to catalyze an array of chemical reactions involving various PUFA (from C_18_ to C_22_) with triple ethylenic bonds [49]. Despite representing only a minor LOX isoform, the PhLOX protein was shown to possess unique catalytic properties responsible for the production of several downstream volatiles, presumably implicated in defense strategies of red algae in the marine ecosystem [62]. Pyropia LOX gene groups, along with those of other red algae, were concluded to have separated from the ancestor of higher plant and animal LOX clades in the early stages of evolution and that might be evolved after horizontal gene transfer from the Gram-negative marine bacterium *Shewanella violacea* DSS12 [62].

#### 2.1.1. Rhodophyta

Red algae have provided interesting models to investigate the evolution of the fatty acid metabolism and the function of oxylipins in photosynthetic organisms [63]. Among the different algal phyla, Rhodophyta has been, in fact, the most prolific source of oxylipins, predominantly as result of widespread LOX metabolism, in which C_20_ PUFA, namely eicosapentaenoic (**4**) and arachidonic (**6**) acids, as well as C_18_ PUFA (linoleic (**1**) and α-linolenic (**2**) acids), are employed as substrates. The majority of oxylipin structures characterized so far in red macroalgae comes from the metabolism of C_20_ PUFA via 12-LOX activity. Nevertheless, other enzymes, including arachidonate 5*R*-, 8*R*-, and 15*S*-LOX, as well as linoleate 9*S*- and 13*S*-LOX, were detected in red algae [3,26].

Kumari *et al.* [24] assessed the content of nine different endogenous hydroxy-oxylipins in forty species belonging to the three algal phyla. Among Rhodophyta, the total oxylipin content ranged from 19.4 ± 2.2 (*Laurencia cruciata* Harvey) to 1,753.1 ± 268.2 ng/g (*Gracilaria corticata* v. *folifera*), fresh weight [24]. Despite the large variability observed, which could be attributed to the availability of their substrate fatty acids, species-specific LOX activity, or to other factors, the red macroalgae showed to be particularly rich sources of hydroxyeicosatetraenoic acids [24].

**Figure 3:**
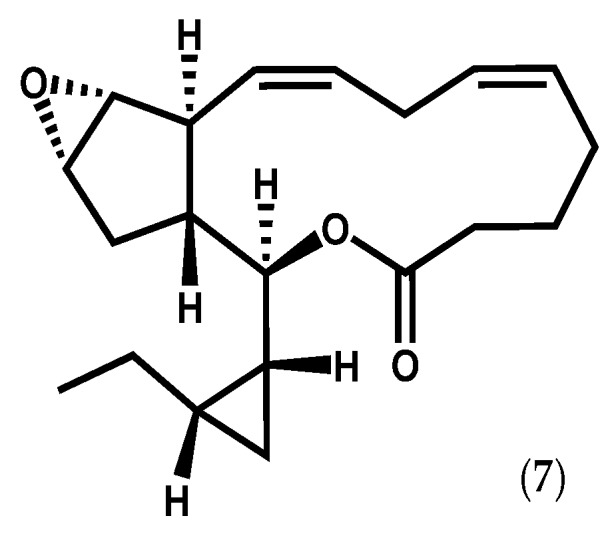
Chemical structure of hybridalactone.

One of the earlier reports of novel oxylipins involved *Laurencia hybrida* (A.P. de Candolle) T. Lestiboudois, from which hybridalactone (**7**, Figure 3), the first marine-derived oxylipin containing a cyclopropane and a macrolactone ring, was isolated [64]. The structure and relative/absolute configuration of this complex oxylipin was elucidated by spectroscopic methods, including X-ray diffraction, molecular mechanics calculations, chemical derivation, and total synthesis [64,65,66,67,68]. Along with hybridalactone (**7**), Higgs [64] also reported the structure of another fatty acid derivative in *L. hybrida*, originally assigned as 9-hydroxyeicosapentaenoic acid; however, its structure was corrected years later by comparison of its spectral data with those of 12*S*-hydroxyeicopentaenoic acid (12*S*-HEPE) (**8**, Figure 2d) from *Murrayella periclados* (C. Agardh) F. Schmitz [69]. The occurrence of 12*S*-HEPE (**8**) in *L. hybrida* supports its intermediacy in the biogenesis of hybridalactone (**7**) via 12-LOX [69].

**Figure 4:**
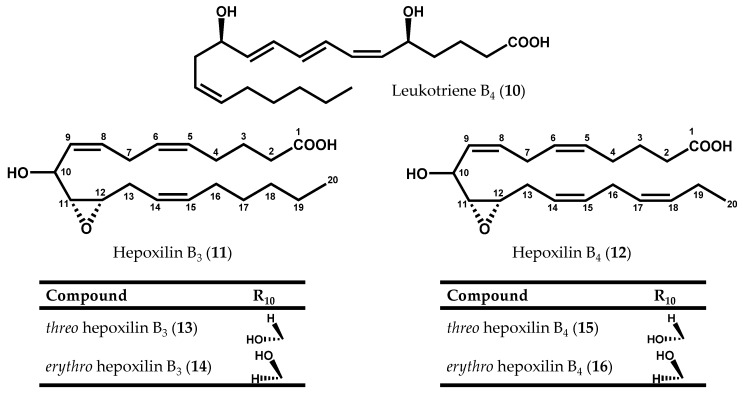
Chemical structures of leukotriene B_4_, and hepoxilins B_3_ and B_4_ diastereoisomers.

Soon afterwards, 12*S*-HEPE (**8**) was also detected in other two red algae species, *Platysiphonia miniata* (C. Agardh) Børgesen [70] and *Gracilariopsis lemaneiformis* (Bory de Saint-Vincent) E.Y. Dawson, Acleto & Foldvik [71]. In addition to 12*S*-HEPE (**8**), Bernart & Gerwick [72] isolated 12*S*-hydroxyeicosatetraenoid acid (12*S*-HETE) (**9**, Figure 2f) and leukotriene B_4_ (**10**), as well as a mixture of hepoxilin B_3_ (**11**) and B_4_ (**12**) diastereomers (**13-16**) from *M. periclados* (Figure 4) [72]. Among *M. periclados* metabolites, 12*S*-HEPE (**8**) displayed potentially useful biological activities, including inhibition of phospholipase A_2_ (PLA_2_) (IC_50_ = 22 µM) and a similar inhibition of both Na^+^/K^+^ ATPase (IC_50_ = 30 µM) and H^+^/K^+^ ATPase (IC_50_ = 30 µM) [72]. Nevertheless, the discovery of leukotriene B_4_ (**10**) represented, perhaps, the most striking parallelism between marine and mammalian fatty acid metabolism. Another fatty acid derivative common to human metabolism, hepoxilin B_3_ (**11**), was previously identified in *P. miniata* and *Cottoniella filamentosa* (M.A. Howe) Børgesen [73]. In humans, hepoxilins are known to act on plasma permeability on skin, to induce a specific-receptor-dependent Ca^2+^ mobilization from endogenous sources, as well as the release of arachidonic acid (**6**) and diacylglycerol [74]. The mammalian biosynthesis of these epoxy-hydroxy eicosanoids has been studied in detail and it may be the result of an intramolecular rearrangement of 12-hydroperoxyeicosatetraenoic acid (12-HpETE) (**17**, Figure 2f) [75,76].

**Figure 5:**
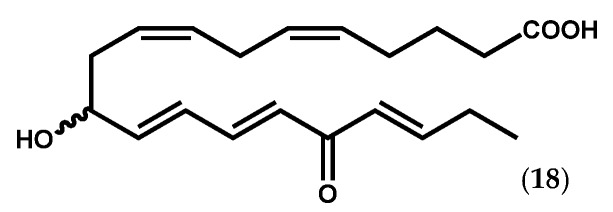
Chemical structure of ptilodene.

Collections of *Ptilota filicina* J. Agardh were the source of new fatty acid derivatives, among which ptilodene (**18**, Figure 5) showed slight antimicrobial activity against pathogenic Gram-negative and Gram-positive bacteria, and acted as an inhibitor against 5-LOX and Na^+^/K^+^ ATPase [77,78].

**Figure 6:**
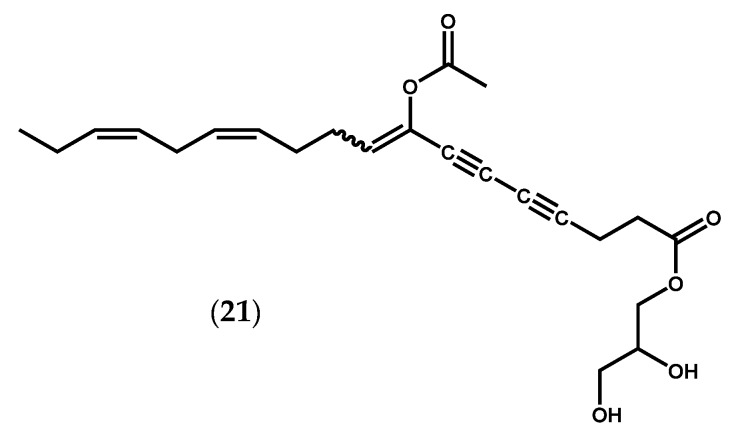
Chemical structure of peyssonenynes A and B.

Other three homologous oxylipins produced by a 12-LOX pathway were isolated from *Farlowia mollis* (Harvey & Bailey) Farlow & Setchell and structurally elucidated by spectrochemical methods [79]. Two of them, 12*R*,13*S*-dihydroxyeicosatetraenoic acid (12*R*,13*S*-diHETE) (**19**, Figure 2f) and 12*R*,13*S*-dihydroxyeicosapentaenoic acid (12*R*,13*S*-diHEPE) (**20**, Figure 2d), as well as other oxylipins, were also detected in *G. lemaneiformis* [16,71,80]. Hamberg & Gerwick [80] reported the presence of additional enzymatic activity, other than LOX, in *G. lemaneiformis*, an AOS that catalyzes the conversion of 12-HpETE (**17**) into 12*R*,13*S*-diHETE (**19**) [80]. The two vicinal diol-fatty acids 12*R*,13*S*-diHETE (**19**) and 12*R*,13*S*-diHEPE (**20**) also displayed interesting biological properties, including inhibition of 5-LOX in A23187-stimulated human polymorphonuclear leukocytes (38% inhibition at 10^−4^ M) and of dog kidney Na^+^/K^+^ ATPase (54% inhibition at 10^−4^ M) [79]. Other remarkable biological effects have been described for unique oxylipins. Peyssonenynes A and B (**21**, Figure 6), firstly isolated in 2004 from the Fijian red marine alga *Peyssonnelia caulifera* Okamura, were roughly equipotent on *in vitro* inhibition of DNA methyl transferase 1 (DNMT1) (IC_50_ values of 16 and 9 µM, respectively) [81]. Selective DNMT inhibitors might rapidly reactivate the expression of epigenetically-silenced tumor suppressor genes, and this reactivation could lead to growth inhibition of tumor cells or alteration of their sensitivity to other anticancer therapies. Two DNMT inhibitors (5-aza-cytidine, Vidaza^®^, 5-aza-2′-deoxycytidine, Dacogen^®^) are already in use for the treatment of myelodysplastic syndrome. However, these drugs are cytotoxic azanucleosides, and novel inhibitors with alternative mechanisms of action are actively sought [82]. Once peyssonenynes (**21**) are rare in red algae, researchers have already accomplished their total synthesis and other functional assays are being conducted [82,83].

**Figure 7:**
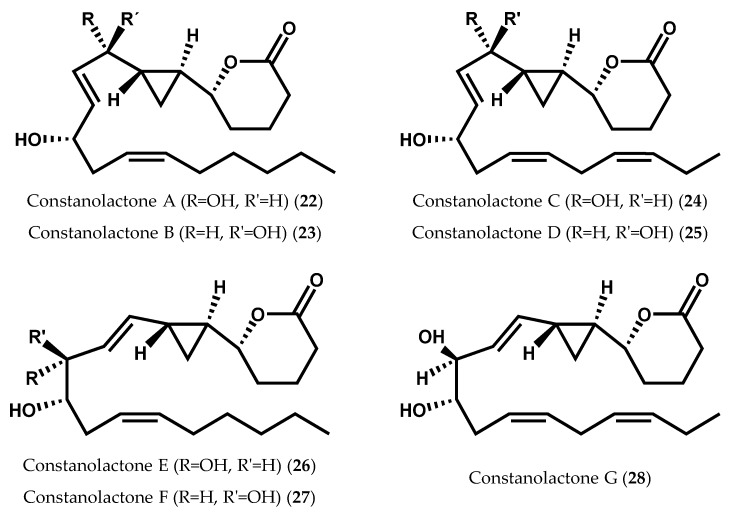
Chemical structures of constanolactones A-G.

Continued isolation efforts yielded lactonized cyclopropyl oxylipins, known as constanolactones (**22**–**28**), from the red marine alga *Constantinea simplex* Setchell (Figure 7) [52,84]. The co-occurrence of other known 12-LOX metabolites, 12*S*-HETE (**9**) and 12*S*-HEPE (**8**), envisions, again, a 12-LOX initiated biosynthesis [52,84].

In more recent years, Bouarab *et al.* [26] demonstrated that, when challenged by pathogen green algal endophyte *Acrochaete operculata* J.A. Correa & R. Nielsen extracts, the resistant haploid phase of the marine red alga *Chondrus crispus* Stackhouse produced both C_20_ and C_18_ oxylipins, including 12-HpETE (**17**) and 13-hydroperoxyoctadecadienoic acid (13-HpODE) (**29**, Figure 2a), respectively. Several enzymes related to oxidative lipid metabolism, including LOX, were upregulated in *C. crispus* gametophytes, 24 h following challenge with *A. operculata* elicitors. Therefore, and based on the well-established functional roles of oxylipins in animals and higher plants, Bouarab *et al.* [26] hypothesized that these compounds appear as essential intermediates in the innate immunity of this alga [26]. Likewise, Gaquerel *et al.* [29] demonstrated that, upon linear uptake of methyljasmonate into the tissues of *C. crispus*, a cascade of PUFA oxidation, leading to the synthesis and accumulation of 13-hydroxyoctadecadienoic acid (13-HODE, **30**) and 13-oxo-octadecadienoic acid (13-oxo-ODE, **31**), was triggered (Figure 2a) [29]. From a pharmacological point of view, 13-HODE (**30**) has been described to play a role in modulating cutaneous hyperproliferation and in the suppression of the activity of the epidermal protein kinase C (PKC)-β [85]. Besides LOX activity, the presence of a new enzyme catalyzing the regio- and stereoselective bisallylic (ω-7)-hydroxylation of PUFA from C_18_ to C_22_ was also described [29]. A similar activity was previously found in the crude protein extract from the red alga *Lithothamnion corallioides* [86,87].

A recent study conducted by Kumari *et al.* [88] demonstrated the effects of methyljasmonate in the thalli of *Gracilaria dura* (C. Agardh) J. Agardh. Although the occurrence of methyljasmonate in macroalgae is still not clear, it is widely presumed that, analogously to higher plants, this active form of jasmonic acid regulates a plethora of developmental and stress responses. In fact, methyljasmonate revealed to be a strong elicitor of ROS production in *G. dura* thalli, leading to the induction of a fatty acid oxidation cascade, which resulted in dose- and time-dependent synthesis and accumulation of several hydroxy-oxylipins, as well as in the upregulation of 13-LOX pathway [88].

The analysis of *Polyneura latissima* (Harvey) Kylin extracts revealed the presence of 9*S*-LOX-derived oxylipins, including 9*S*-hydroxyeicosatetraenoic acid (9*S*-HETE, **32**) and other secondary products, such as 9,15-dihydroxyeicosatetraenoic acid (9,15-diHETE, **33**) (Figure 2f) [21]. The authors of this study hypothesized that 9*S*-HETE (**32**) could be the result of a simple peroxidase-type reduction of 9*S*-hydroperoxyeicosatetraenoic acid (9*S*-HpETE) (**34**, Figure 2f). Other rearrangements of 9*S*-HpETE (**34**), possibly catalyzed by an AOS, may yield hepoxilin-like metabolites, and the occurrence of 9,15-diHETE (**33**) suggests the involvement of a second 15-LOX [21].

Moreover, the occurrence of a functional 5*R*-LOX was inferred upon analysis of lipid extracts of *Rhodymenia pertusa* (Postels & Ruprecht) J. Agardh, from which 5-hydroxyeicosatetraenoic acid (5-HETE) (**35**, Figure 2f) and 5-hydroxyeicosapentaenoic acid (5-HEPE) (**36**, Figure 2d), as well as two vicinal diol-fatty acids, 5,6-dihydroxyeicosatetraenoic acid (5,6-diHETE) (**37**, Figure 2f) and 5,6-dihydroxyeicosapentaenoic acid (5,6-diHEPE) (**38**, Figure 2d), were isolated [89].

**Figure 8:**
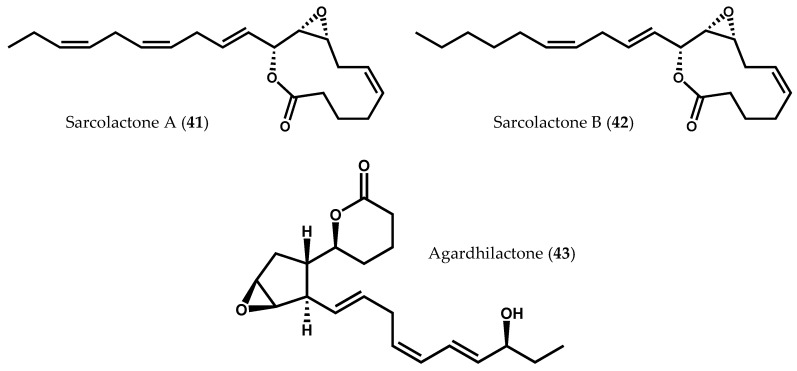
Chemical structures of sarcolactones A and B, and agardhilactone.

Examples of 8-LOX activity have also been described in several species of Rhodophyta, including *Sarcodiotheca gaudichaudii* (Montagne) P.W. Gabrielson [90], *Agardhiella subulata* (C. Agardh) Kraft & M.J. [91], *Gracilaria chilensis* C.J. Bird, McLachlan & E.C. Oliveira [27], and *Gracilaria vermiculophylla* (Ohmi) Papenfuss [31]. Besides the presence of common fatty acid derivatives, such as 8-hydroxyeicosatetraenoic acid (8-HETE, **39**) and 7,8-dihydroxyeicosatetraenoic acid (7,8-diHETE, **40**), some unique carbocyclic oxylipins with an 8-LOX precedence were also identified (Figure 2f) [31,90,91]. Two members of the same family (Solieriaceae), *S. gaudichaudii* and *A. subulata,* provided sarcolactones A (**41**) and B (**42**) [90], and agardhilactone (**43**) [91], respectively, from 8-LOX metabolism (Figure 8). Sarcolactone A (**41**) and agardhilactone (**43**) are closely related metabolites, envisioning a common intermediate for these carbocyclic oxylipins [91]. Nylund *et al.* [31] isolated 8-HETE (**39**) and 7,8-diHETE (**40**), as well as novel conjugated lactones and traces of leukotriene B_4_ (**10**), from mechanically wounded tissues of *G. vermiculophylla* [31]. Lion *et al.* [27] had previously studied the response of *G. chilensis* (a noninvasive alga closely related to the invasive species *G. vermiculophylla*) to wounds, reporting the production and release of 8-HETE (**39**) and 7,8-diHETE (**40**) after tissue disruption [27]. The upregulation of these oxylipins, particularly of 7,8-diHETE (**40**), in wounded algae suggests that both *G. vermiculophylla* and *G. chilensis* respond similarly to tissue damage. The two algae species rely then on a conserved defense mechanism (rapid LOX-mediated transformation of arachidonic acid (**6**) to structurally diverse oxylipins against herbivory), the invasive potential of *G. vermiculophylla* being partly explained by the exclusive detection of prostaglandins in this species [33].

**Figure 9:**
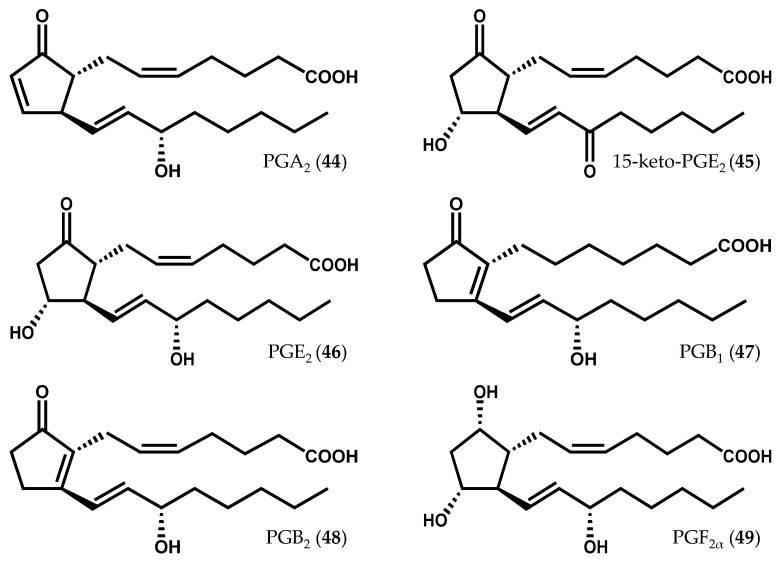
Chemical structures of some prostaglandins described in red algae.

The occurrence of prostaglandins (Figure 9) was primarily reported in the *Gracilaria* genus; however, prostaglandin A_2_ (PGA_2_, **44**) and 15-keto-PGE_2_ (**45**), a stable derivative of PGE_2_ (**46**), were also synthesized *in vivo* by *C. crispus* gametophytes treated with 50 or 100 µM methyljasmonate for 6 h [29]. In a previous study, PGB_1_ (**47**) and PGB_2_ (**48**) had not been detected *in vivo,* but their presence was evidenced in elicited *C. crispus* upon incubation with arachidonic acid (**6**) [26]. Gregson *et al.* [92] reported, for the first time, the presence of PGE_2_ (**46**) and PGF_2α_ (**49**) in *Gracilaria lichenoides* (J.V. Lamouroux) Greville [92]. Afterwards, other prostaglandins, including PGA_2_ (**44**), PGE_2_ (**46**), and 15-keto-PGE_2_ (**45**) were identified in both *Gracilaria verrucosa* (Hudson) Papenfuss [93,94,95] and *G. vermiculophylla* [31,33], along with other fatty acid derivatives. While the prostaglandin-endoperoxide pathway of prostaglandin biosynthesis in invertebrate marine animals has been demonstrated in corals [96,97,98] and in crustaceans [99], the prostaglandin biosynthetic pathway of non-animal organisms has remained unknown for years. Kanamoto *et al.* [100] have identified the first non-animal prostaglandin endoperoxide H synthase (PGHS) gene in the alga species *G. vermiculophylla* and cloned it in a prokaryotic expression system for the production of PGF_2α_ (**49**) [100]. Varvas *et al.* [101] further characterized the structure and function of *G. vermiculophylla* PGHS, concluding that this enzyme displays atypical structural and catalytic features [101].

Altogether, marine red algae are well documented to contain a variety of oxylipins of pharmacological interest and with important biological functions in algae biology, especially, as signaling molecules following stress responses that may regulate algae innate immunity.

#### 2.1.2. Ochrophyta

Ochrophyta has emerged as a source of structurally unique oxylipins derived from novel pathways. As in Rhodophyta, both C_18_ and C_20_ PUFA are employed as substrates for LOX and other enzymatic systems, such as HPL [2]. The brown macroalgae, particularly those of the order Laminariales, also known as kelps, have been mainly reported to exhibit arachidonate 12- and 15-LOX activities, which catalyze the formation of a number of hydroxylated fatty acid derivatives, short chain aldehydes, and carboxylic oxylipins.

**Figure 10:**
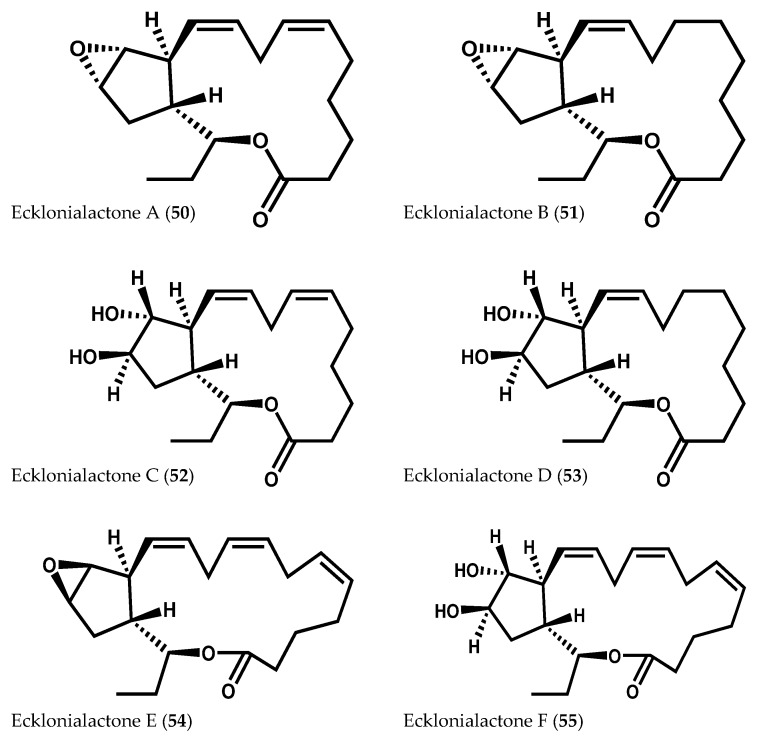
Chemical structures of ecklonialactones A–F.

Ecklonialactones A (**50**) and B (**51**), C_18_ tricyclic compounds, were initially isolated from the brown alga *Ecklonia stolonifera* Okamura as metabolites with invertebrate antifeedant activity against the abalone *Haliotis discus hannai* Ino (Figure 10) [102]. Later, Kurata and co-workers [103] found another four ecklonialactones (C–F (**52**–**55**)) in *E. stolonifera* (Figure 10) [103]. These same metabolites, along with other related classes of cyclical oxylipins, were also described in *Cymathaere triplicata* (Postels & Ruprecht) J. Agardh [23,90,104], *Egregia menziesii* (Turner) Areschoug [19], *Laminaria sinclairii* (Harvey ex J.D. Hooker & Harvey) Farlow, Anderson & Eaton [105], and *Eisenia bicyclis* (Kjellman) Setchell [22].

**Figure 11:**
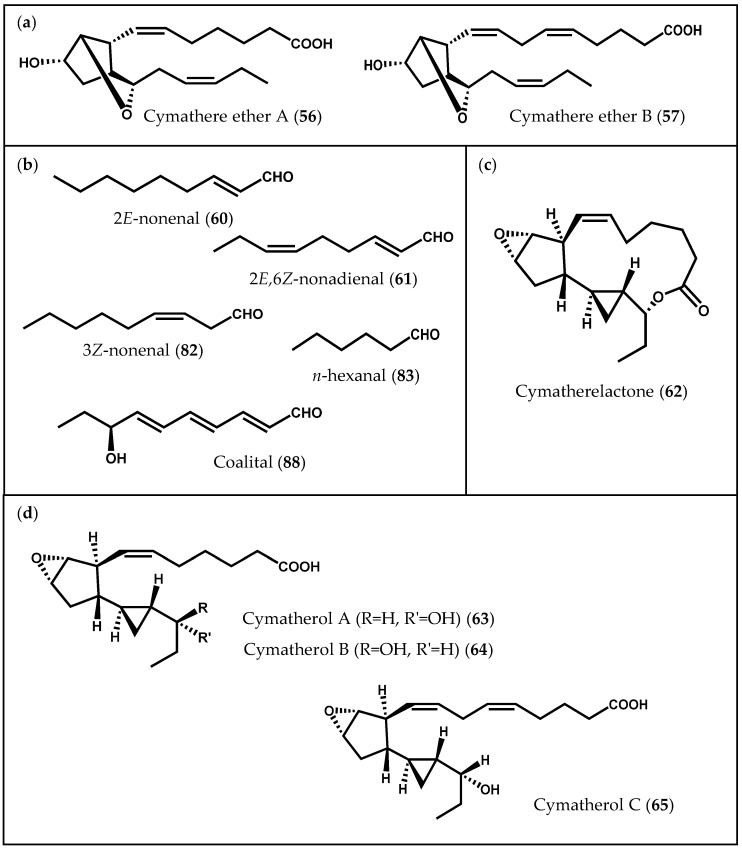
Chemical structures of (**a**) cymathere ethers A and B; (**b**) aldehydes; (**c**) cymatherelactone; and (**d**) cymatherols A–C.

In addition to simpler hydroxylated fatty acids, a series of prostanoid-like metabolites has been isolated from the edible kelp *C. triplicata*. Proteau and Gerwick [104] began by reporting two bicyclic oxylipins, cymathere ethers A (**56**) and B (**57**) (Figure 11a), and proposed 10*S*-hydroxyoctadecatetraenoic acid (10*S*-HODTA) (**58**, Figure 2c) as a biosynthetic intermediate [104]. Further experiments led to the isolation of unique molecules from the extract of *C. triplicata*, as well as hydroxylated fatty acids: 12*S*-HETE (**9**), 13*S*-hydroxyoctadecatetraenoic acid (13*S*-HODTA) (**59**, Figure 2c), and 10*S*-HODTA (**58**). Likewise, hydroperoxide cleavage products, such as 2*E*-nonenal (**60**) and 2*E*,6*Z*-nonadienal (**61**), responsible for the characteristic odor of fresh alga and believed to be physiologically active, were also found in *C. triplicata* extracts (Figure 11b) [90]. The elucidation of cymatherelactone (**62**, Figure 11c) and cymatherols A–C structures (**63**–**65**, Figure 11d) was then accomplished by a combination of spectroscopic techniques and synthetic derivatization [23]. These new oxylipins contain cyclopentyl, cyclopropyl, epoxyde, and lactone rings, and all of them are likely to be synthesized from the C_18_ PUFA stearidonic acid (**3**), with the exception of cymatherol C (**65**), which is predicted to derive from eicosapentaenoic acid (**4**). Moreover, the sodium channel modulating properties of the isolated compounds were evaluated using Neuro-2a cell line: cymatherelactone (**62**) exhibited moderate sodium channel blocking activity (IC_50_ = 16 µM) [23].

**Figure 12:**
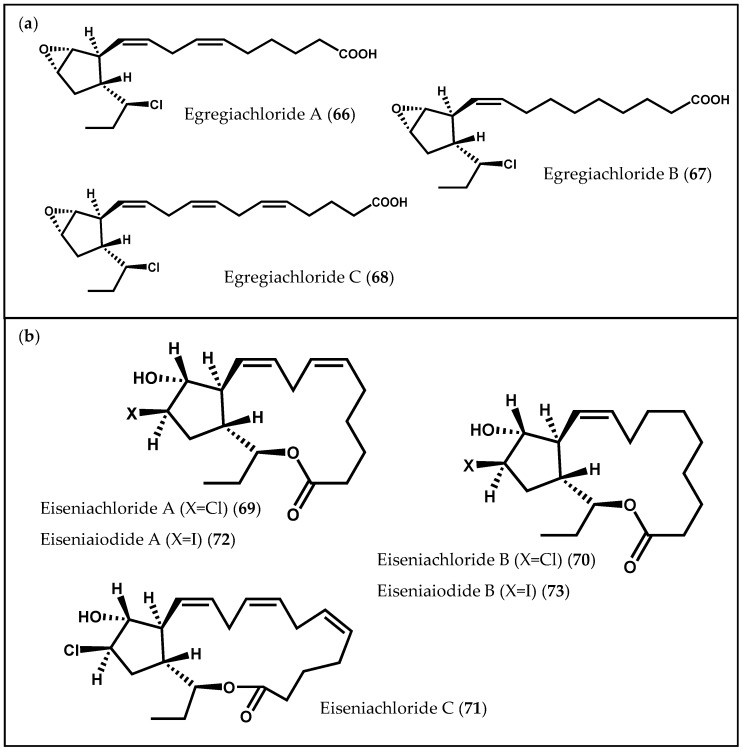
Chemical structures of (**a**) egregiachlorides and of (**b**) eiseniachlorides and eiseniaiodides.

Another class of cyclic oxylipins, the egregiachlorides (Figure 12a), was found for the first time in *E. menziesii,* along with ecklonialactones A (**50**), B (**51**) and E (**54**), previously described [106]. Egregiachlorides A–C (**66**–**68**) are characterized by a cyclopentyl ring with an adjacent chlorine functionality [106]. The occurrence of egregiachlorides A (**66**) and B (**67**) was later observed in the brown alga species *E. bicyclis* [22]. Besides these chlorinated C_18_ oxylipins, cymathere- and lactone-type oxylipins, as well as novel ecklonialactone derivatives containing either a chloride (eiseniachlorides (**69**–**71**)) or an iodide atom (eiseniaiodides, **72** and **73**) (Figure 12b), were also described in *E. bicyclis* [22]. The biogenesis of these metabolites is likely to involve the oxidation of a C_18_ PUFA catalyzed by 13-LOX, leading to the formation of a 13-hydroperoxide compound, which subsequently undergoes a number of rearrangements. Moreover, Kousaka *et al.* [22] evaluated the antibacterial capacity of the isolated oxylipins against two bacterial strains (*Bacillus subtilis* Cohn and *Staphylococcus aureus* Rosenbach). The halogenated oxylipins displayed a moderate inhibition against both bacteria [22].

Besides the great diversity of structurally unique and complex oxylipins, Ochrophyta also contains several known hydroxylated fatty acid derivatives. Initial studies explored the oxylipin chemistry of three brown algae species of the genus *Laminaria*: *Laminaria sinclairii* (Harvey ex J.D. Hooker & Harvey) Farlow, Anderson & Eaton, *Laminaria saccharina* (Linnaeus) J.V. Lamouroux, and *Laminaria setchellii* P.C. Silva [18]. These species were found to contain 13*S*-HODTA (**59**), 13*S*-hydroxyoctadecadienoic (13*S*-HODE) (**74**, Figure 2a), and 13*S*-hydroxyoctadecatrienoic (13*S*-HOTrE) (**75**, Figure 2b), as well as 15*S*-hydroxyeicosatetraenoic (15*S*-HETE) (**76**, Figure 2f), and 15*S*-hydroxyeicosapentaenoic (15*S*-HEPE) (**77**, Figure 2d) acids, suggesting 15-LOX activity [18]. In terms of biological activity, 13*S*-HODE (**74**) has been shown to induce apoptosis in colorectal cancer cells by down-regulation of peroxisome proliferator-activated receptor (PPAR)-δ [107], also exhibiting remarkable tumor necrosis factor (TNF)-α inhibitory activity (52% and 98% inhibition at 50 µM and 100 µM, respectively) [60]. Additionally, previous studies demonstrated that 15*S*-HEPE (**77**) inhibits the growth and the production of arachidonic acid (**6**)-derived metabolites in human prostatic cancer cells, presumably by PPAR-γ activation [108].

**Figure 13:**
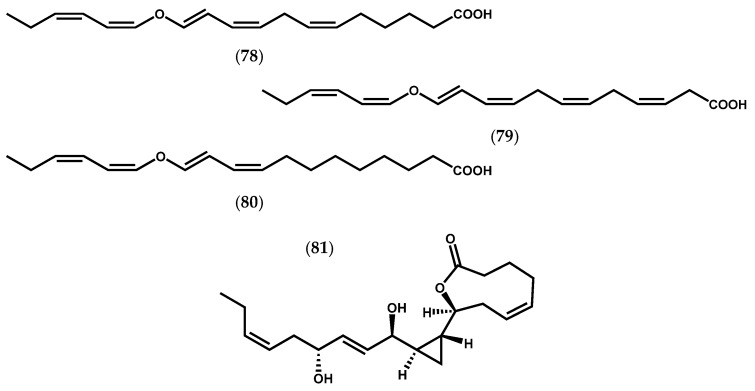
Chemical structures of divinyl ether-fatty acids from *L. sinclairii*, and neohalicholactone.

Moreover, three divinyl ether-fatty acids (**78**–**80**, Figure 13) were found in *L. sinclairii*, which are indicative of a LOX with ω6 specificity. Later, the analysis of an extract from *L. sinclairii* led to the isolation of neohalicholactone (**81**, Figure 13), a cyclopropyl-containing oxylipin firstly isolated from the marine sponge *Halichondria okadai* Kadota [105].

Several hydroperoxides deriving from a LOX-catalyzed oxygenation of arachidonic acid (**6**) were detected in the edible species *Laminaria angustata* Kjellman [109,110]. These LOX-derived fatty acid hydroperoxides were found to be the intermediate products of C_6_ and C_9_ aldehyde formation via the action of HPL. Boonprab and co-workers [109] showed that *L. angustata* produces C_9_ aldehydes, namely 3*Z*-nonenal (**82**) and 2*E*-nonenal (**60**), exclusively from C_20_ PUFA, whereas the C_6_ aldehyde *n*-hexanal (**83**) derives either from C_18_ or from C_20_ fatty acids (Figure 11b) [109]. Similarly to higher plants, these short-chain aldehydes appear to exert vital functions in chemical attraction and defense [6,110].

A growing body of evidence has been supporting the pivotal role of different oxylipins in defense induction of marine brown algae. Küpper *et al.* [28] found that bacterial lipopolysaccharides can be strong triggers of early events of defense reactions in the kelp species *Laminaria digitata* (Hudson) J.V. Lamouroux. It was shown that the challenge of *L. digitata* sporophytes resulted in an oxidative burst and the rapid release of free saturated and unsaturated fatty acids, with concomitant accumulation of oxylipins, such as 13-hydroxyoctadecatrienoic (13-HOTrE) (**84**, Figure 2b) and 15-hydroxyeicosapentaenoic (15-HEPE) (**85**, Figure 2d) acids [28]. The latter was found to inhibit the production of proinflammatory mediators in rat basophil leukemia (RBL)-1 cells [85].

Later, Küpper *et al.* [30] demonstrated that free PUFA, as well as methyljasmonate, were responsible for triggering oxidative burst in young *L. digitata* sporophyte thalli, which consequently activated a range of downstream signaling events, including fatty acid oxidation pathways [30]. Further studies evidenced that PGA_2_ (**44**) was able to induce a more powerful oxidative burst than the response triggered by most of the chemical elicitors in *L. digitata*. However, rather few effects at other levels of signal transduction were observed. PGA_2_ (**44**) did not induce the release of free fatty acids, and only 15-hydroxyeicosatetraenoic acid (15-HETE) (**86**, Figure 2f) was found to be upregulated in *L. digitata* [34].

**Figure 14:**
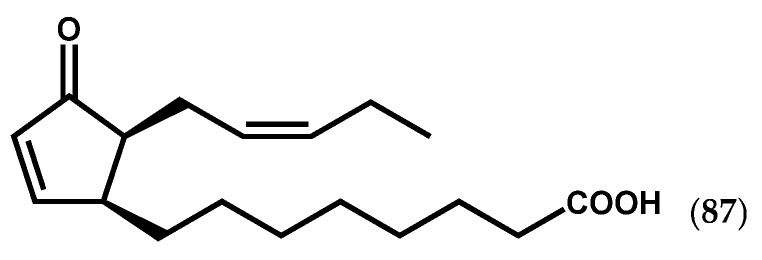
Chemical structure of 12-OPDA.

Until 2010, global molecular analyses of brown algal stress response were hampered by the lack of genomic resources. The access to *Ectocarpus siliculosus* (Dillwyn) Lyngbye genome sequence by Cock *et al.* [111] represented a major breakthrough in algal research. For instance, although the endogenous occurrence and relevance of jasmonates in macroalgae are still unclear, the presence of AOS and AOC genes involved in the initial step of jasmonates’ biosynthesis, in contrast with the absence of genes for jasmonic acid carboxyl methyl transferase in the *Ectocarpus* genome, suggested that (i) jasmonates may not have the same function in brown algae as in land plants or (ii) they have evolved to serve similar functions using different regulatory systems [42,88]. In fact, the accumulation of C_18_ cyclic oxylipins like 12-oxo-phytodienoic acid (12-OPDA) (**87**, Figure 14), the biosynthetic precursor of jasmonates, as well as of a number of C_20_ cyclic prostaglandins was described in *L. digitata* and *E. siliculosus* under copper stress, supporting the role of putative cyclopentenones in the defensive mechanisms of brown algae [40,42].

Kumari *et al.* [24] assessed the content of nine different endogenous hydroxy-oxylipins in seven macroalgae species belonging to Ochrophyta. The total oxylipin contents ranged from 345.4 ± 56.8 (*Scytosiphon lomentaria* (Lyngbye) Link) to 2574.5 ± 155.5 ng/g (*Stoechospermum marginatum* (C. Agardh) Kützing), fresh weight [24]. The presence of oxylipins in a number of species belonging to Ochrophyta suggests that this group of macroalgae offers a potential source of these biologically active fatty acid derivatives. Years before Rorrer *et al.* [112] established cell suspension cultures of *L. saccharina* for the commercial production of hydroxy fatty acids derived from both C_18_ and C_20_ PUFA [112]. However, the low production of algal biomass, along with their poor ability to utilize exogenously supplied PUFA, rendered oxylipin production a failure on a commercial scale [24]. In more recent years, novel methods for oxylipin production from different PUFA were patented [113,114,115].

#### 2.1.3. Chlorophyta

Unlike Rhodophyta and Ochrophyta, studies on the green algal oxylipin chemistry are much scarcer. As members of Chlorophyta are typically rich in C_18_ PUFA, similar trends are expected to reflect for their oxidized derivatives. Besides the action of 9- or 13-LOX already described in green macroalgae, the action of HPL is also characteristic of the oxylipin pathways in this algal phylum, which results in the production of a variety of short-chain carbohydrates, aldehydes, and alcohols [2].

One of the earlier studies exploring oxylipin chemistry in green macroalgae revealed the presence of unprecedented fatty acid derivatives in *Acrosiphonia coalita* (Ruprecht) Scagel, Garbary, Golden & M.W. Hawkes [116]. Bernart *et al.* [116] found that this species was able to produce a wide assortment of oxylipins that are likely to emanate from 9-LOX metabolism of C_18_ PUFA precursors. Additional oxidation may give rise to chain cleaved aldehydes, like coalital (**88**, Figure 11b), which was effective at inhibiting the growth of the pathogenic yeast *Candida albicans* Berkhout, at concentrations as low as 100 μg/disk, using the sensitivity disk assay [116]. Gerwick *et al.* [17] have also found another green alga species (*Cladophora columbiana* F.S. Collins) to be a source of oxylipins, some of which may arise from HPL cleavage of a 9-hydroperoxyoctadecatrienoic acid (9-HpOTrE) (**89**, Figure 2b) precursor, itself formed by the action of a linoleate 9-LOX [17]. 12- and 15-LOX were further identified as the major LOX isoforms in *Enteromorpha intestinalis* (Linnaeus) Nees, yielding 15-HETE (**86**) and 12-hydroxyeicosatetraenoic acid (12-HETE) (**90**, Figure 2f), after arachidonic acid (**6**) treatment [117]. A calcium-stimulated LOX was partially purified from the green alga *Ulva lactuca* Linnaeus [50]. The isolated LOX can cleave different PUFA substrates into a number of hydroxy-fatty acids, including 9-hydroxyoctadecadienoic acid (9-HODE) (**91**, Figure 2a), 13-HODE (**30**), 9-hydroxyoctadecatrienoic acid (9-HOTrE) (**92,** Figure 2b), 12-HETE (**90**), 15-HETE (**86**), 12-hydroxyeicosapentaenoic acid (12-HEPE) (**93**, Figure 2d), and 14-hydroxydocosahexaenoic acid (14-HDoHE) (**94**, Figure 2e) [50]. Later, enantioselective formation of 9*R*-hydroperoxyoctadecadienoic acid (9*R*-HpODE) (**95**, Figure 2a) and 9*R*-hydroperoxyoctadecatrienoic acid (9*R*-HpOTrE) (**96**, Figure 2b), as well as of 11*R*-hydroperoxyeicosatetraenoic acid (11*R*-HpETE) (**97**, Figure 2f) and middle-chain aldehydes were described following C_18_ and C_20_ PUFA incubation with a crude enzyme of *Ulva conglobata* Kjellman [118,119]. These observations strongly suggest the presence of 9- and 11-LOX along with HPL activity in this marine green algal species. Soon afterwards, Tsai *et al.* [120] have immobilized a marine algal 11-LOX from *Ulva fasciata* Delile for potential application by the seafood industry in specific aroma generation [120].

More recently, Kumari *et al.* [24] determined the content of hydroxy-oxylipins in several species of macroalgae, having found that the ones belonging to Chlorophyta contained the highest amounts of these oxidized metabolites (from 141.2 ± 12.2 ng/g fresh weight in *Codium dwarkense* Børgesen to 8161.9 ± 253 ng/g fresh weight in *Chaetomorpha linum* (O.F. Müller) Kützing), particularly octadecanoids. Despite the dominance of C_18_ PUFA, arachidonic acid (**6**)-dependent LOX activity was also found, exhibiting 8-, 12-, and 15-LOX isoforms similar to that of arachidonate 11-LOX activity previously reported in *U. fasciata*, *U. conglobata*, and to arachidonate 12-, and 15-LOX in *E. intestinalis* [24]. The genus *Ulva* has gained worldwide prominence and has emerged as a model for investigating complex metabolic networks, due to its high growth rate and innate ability to grow in wider environmental conditions. In this regard, lipidomic and biochemical changes induced by various stress conditions have been investigated in the species *U. lactuca* [39,46,47]. For instance, this intertidal alga was able to cope with nitrate and phosphate nutritional stress by altering the metabolic pathways involved in lipid biosynthesis, including a shift in lipid classes, fatty acids, and oxylipins [47]. The alteration of lipid content is known to be one of the most important adaptation strategies to nutrient imbalance in macroalgae. The increased availability of PUFA in nutrient-supplemented *U. lactuca* thalli led to an increased LOX activity, concomitant with the increase in hydroxy-oxylipin compounds, which have already exhibited defensive roles against oxidative stress conditions in macroalgae [39,46]. The increase in LOX activity and in the relative contents of several hydroxyoctadecadienoic, hydroxyoctadecatrienoic and hydroxyeicosatetraenoic acids suggest the upregulation of different enzyme isoforms, including linoleate 9-LOX, linolenate 13-LOX, as well as arachidonate 5-, 8-, 12-, and 15-LOX [47]. In contrast, the levels of hydroperoxy-oxylipins decreased after nutrient supplementation, pointing to a ROS-mediated nonenzymatic lipid peroxidation due to nutritional limitation-induced oxidative stress.

### 2.2. Nonenzymatically-Derived Algal Oxylipins: The Phytoprostanes

Before it became possible for enzymatic oxylipin signaling pathways to evolve, another reaction sequence that gives rise to a great variety of oxylipins was already present in all aerobic PUFA-containing organisms: free-radical-catalyzed nonenzymatic lipid peroxidation. This early chemical process, which has prevailed throughout the evolution of the oxylipin pathways, can be catalyzed by ROS, which are generated continuously during normal aerobic metabolism [121]. However, a massive production of ROS can likely represent a hallmark of defense responses to a variety of abiotic and biotic stresses. Nonenzymatic reactions are therefore widespread in organisms, even in healthy ones, and because they often evade genetic studies, their relevance can be difficult to estimate. Nonenzymatic lipid oxidation is usually viewed as deleterious; however, recent evidence suggests that during stress, both lipid peroxidation and reactive electrophile species (RES) generation can eventually benefit cells [122].

Phytoprostanes are the resulting products of the autoxidation of α-linolenic acid (**2**), one of the most abundant PUFA in terrestrial plant membranes, being also present in macroalgae. So far, most studies focused on the nonenzymatically-derived oxylipins from higher terrestrial plants and information regarding the occurrence of this large family of biologically active oxidized lipids in macroalgae is still scarce. However, the presence of α-linolenic acid (**2**), the known precursor of phytoprostanes, in macroalgae, along with the broad fluctuations of environmental conditions that characterize the marine ecosystem, suggest that macroalgae could be valuable sources of phytoprostanes [123].

**Figure 15:**
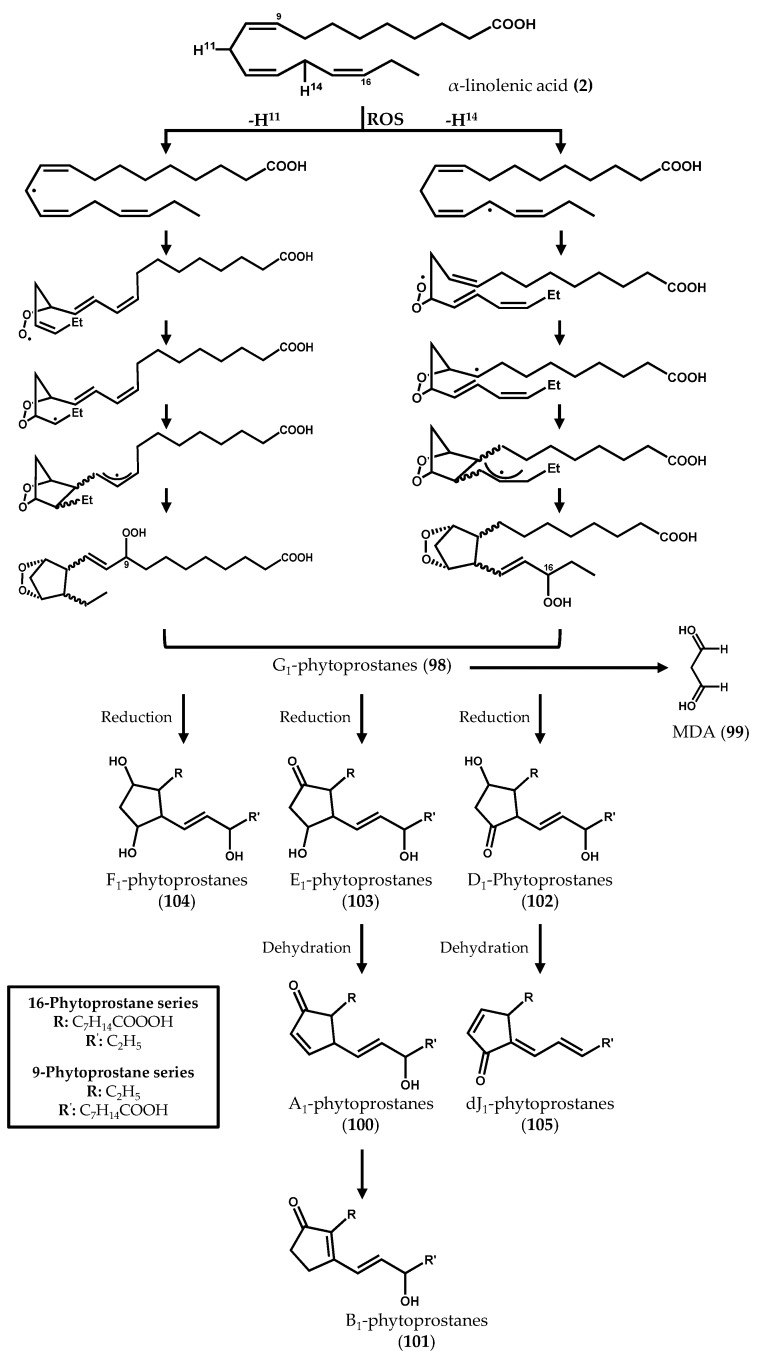
Nonenzymatic formation of phytoprostanes from α-linolenic acid.

The biosynthesis of phytoprostanes (Figure 15) is proposed to be initiated by the attack of ROS to α-linolenic acid (**2**), yielding a linolenate radical that readily oxidizes and cyclizes to complex regio- and stereoisomeric prostaglandin-like compounds [124]. Two regioisomeric series (16- and 9-series) can be generated according with the position where the hydrogen abstraction occurs and the oxygen atoms are inserted into the PUFA backbone [125]. G_1_-phytoprostanes (**98**) can spontaneously decay, forming malondialdehyde (MDA) (**99**), or be the precursors of different classes of phytoprostanes, named in analogy with the prostaglandin nomenclature system as A_1_ (**100**), B_1_ (**101**), D_1_ (**102**), E_1_ (**103**), F_1_ (**104**), dJ_1_ (**105**), and L_1_ (**106**) phytoprostanes, the latter being the regioisomer of B_1_-phytoprostanes (**101**) [126]. Thus, a myriad of oxygenated lipids is generated, some of which remain anchored in membranes, while others are released.

Ritter *et al.* [42] have recently described the accumulation of A_1_-phytoprostanes (**100**) in the brown macroalgae *E. siliculosus* subjected to copper stress, thus supporting the occurrence of ROS-mediated lipid peroxidation processes [42]. These results also suggest the involvement of phytoprostanes in macroalgae defense responses. In fact, previous reports in land plants have shown that phytoprostanes exert a wide range of biological activities, inducing, for instance, the biosynthesis of secondary metabolites, the expression of genes involved in detoxification processes, and the regulation of the oxidative stress-related mitogen-activated protein kinase (MAPK)-dependent signaling pathway [127,128,129]. Despite these observations, the exact role and physiological function of phytoprostanes have not been yet fully elucidated.

**Figure 16:**
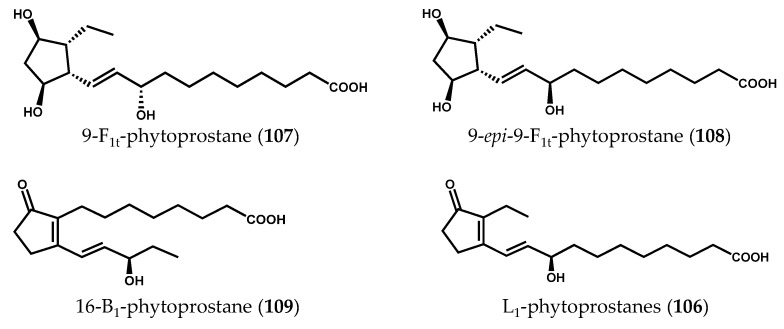
Chemical structures of naturally occurring free phytoprostanes in macroalgae.

In a recent study conducted by our research group [123] the naturally occurring free phytoprostane composition of 24 macroalgae species was determined, using a fast, selective, and robust ultrahigh-performance liquid chromatography coupled to triple-quadrupole mass spectrometry (UHPLC-QqQ-MS/MS) method. The analysis of phytoprostanes in natural matrices is extremely challenging, requiring highly sensitive and specific tools for their profiling and characterization [130]. Additionally, the great diversity granted by the presence of racemic mixtures of phytoprostanes increases the complexity of these analyses. The phytoprostane qualitative and quantitative profiles varied greatly among all macroalgae samples (Figure 16), F_1t_-phytoprostanes, comprising both 9-F_1t_-phytoprostane (**107**) and 9-*epi*-9-F_1t_-phytoprostane (**108**), being the dominant class, and L_1_-phytoprostanes (**106**) the minor one. The brown alga species *Cladostephus spongiosus* (Hudson) C. Agardh and the green alga *Codium tomentosum* Stackhouse exhibited higher diversity of compounds, containing 9-F_1t_-phytoprostane (**107**), 9-*epi*-9-F_1t_-phytoprostane (**108**), 16-B_1_-phytoprostane (**109**) and L_1_-phytoprostanes (**106**). The brown alga *Bifurcaria bifurcata* R. Ross presented the lowest total phytoprostane contents (5.68 ± 1.09 ng/100 g, dry algae), whereas *Saccharina latissima* (Linnaeus) C.E. Lane, C. Mayes, Druehl & G.W. Saunders cultivated in an integrated multitrophic aquaculture (IMTA) system was the richest sample (1,380.90 ± 103.83 ng/100 g dry algae). However, no conclusion regarding the advantages of IMTA systems could be drawn, as no marine counterpart of this species was analyzed. Moreover, no correlation between the amount of α-linolenic acid (**2**) in macroalgae material and total phytoprostane content was found, and no phylogenetic relationship was established. Altogether, the collected data suggested that the variations observed in terms of phytoprostane composition could be partially explained by intrinsic factors (e.g., physiological variations within algae organs) and/or extrinsic factors (e.g., geographical origin or area of cultivation, seasonal and environmental variations, time of harvest, water temperature, salinity levels, and processing methods) [123].

Currently, the interest in phytoprostanes targets two general areas: their use as biomarkers of oxidative stress in plant-derived foodstuffs and as bioactive mediators with potential benefits in different biological systems. Evidence points to the involvement of certain phytoprostane classes in the regulation of immune function in humans. E_1_-phytoprostanes (**103**), previously identified in pollen, inhibited dendritic cell interleukin-12 (IL-12) production and increased T helper type 2 (Th2) cell polarization *in vitro* [131]. In contrast, Guttermuth *et al.* [132] found that both E_1_ (**103**) and F_1_ (**104**) phytoprostanes partially inhibited Th1 and Th2 cytokine production *in vivo* [132]. The immunomodulatory effects of E_1_-phytoprostanes (**103**) were found to occur via peroxisome proliferator-activated receptor (PPAR)-γ and nuclear factor-κB (NF-κB)-dependent mechanisms [133,134]. Karg and co-workers [135] reported that A_1_ (**100**) and dJ_1_ (**105**) phytoprostanes displayed anti-inflammatory effects in human embryonic kidney (HEK) cells and RAW264.7 murine macrophages, by down-regulating NF-κB and inhibiting nitric oxide (NO) synthesis, respectively [135]. Recently, Minghetti *et al.* [136] showed that B_1_-phytoprostanes (**101**) were biologically active in experimental models of immature cells of the central nervous system, exhibiting neuroprotective effects against oxidant injury induced by hydrogen peroxide and promoting myelination through PPAR-γ activation [136].

## 3. Conclusions

A large variety of unique oxylipin classes have been found in marine macroalgae, deriving from both developmentally regulated processes (catalyzed by enzymatic systems) and in response to environmental changes (chemical (auto)oxidation). Combined enzymatic and nonenzymatic peroxidation builds the natural peroxide status of membranes. It is the further rearrangement or metabolism of membrane lipid peroxides, by enzymatic and nonenzymatic mechanisms, that results in the accumulation of a far greater variety of secondary oxidation products.

Although oxidized fatty acids are widely distributed in Rhodophyta, Ochrophyta, and Chlorophyta, each major group of algae exhibits its own unique oxylipin signature in terms of fatty acid precursors and typical sites of oxidation. Enzymatically-derived oxylipins from Rhodophyta, most of which result from the metabolism of C_18_ and C_20_ PUFA, have been the most extensively studied. As in red algae, Ochrophyta species use both C_18_ and C_20_ PUFA as substrates for LOX (mostly 13-LOX) and other enzymatic systems. Besides the common oxygenated fatty acid derivatives, both red and brown macroalgae have also revealed a high diversity of unusual and unprecedented oxylipin structures. Studies on oxylipin metabolism in Chlorophyta are much scarcer. Existing data point to a dominance of 9-LOX and HPL activity, resulting in the production of several short chain carbohydrates, aldehydes and alcohols. On the other hand, the occurrence and distribution of algal oxylipins from nonenzymatic reactions is highly unpredictable, differing between species and as a consequence of the surrounding growth conditions.

Macroalgae thrive in a complex seawater environment, being continuously challenged by an array of potentially pathogenic organisms and multivariate ecological changes. Algal oxylipins may then help to control interactions with other organisms and with the environment, promoting algae survival. Besides the eco-physiological role of these oxidized lipid-derivatives and their relevance in macroalgae, there is still a debate on the exact mechanisms of stress tolerance. Moreover, and because metabolites of this class also play a crucial role in both mammalian physiology and disease, interest in the structural chemistry, biosynthesis, and pharmacological activities of these marine products has increased.

All this evidence supports the need for stronger efforts to improve our knowledge of the pathways of oxylipin biosynthesis, their individual role in cellular responses, and the target elements involved in gene regulation, using the combined “omics” approach of genomics, transcriptomics, and metabolomics.

## Abbreviations

The following abbreviations are used in this manuscript:

AOS/AOC: Allele oxide synthase/cyclase
COX: Cycloxygenase
DNMT: DNA methyl transferase
HDoHE: Hydroxydocosahexaenoic acid
HEK: Human embryonic kidney
HEPE: Hydroxyeicosapentaenoic acid
HETE: Hydroxyeicosatetraenoid acid
HODE: Hydroxyoctadecadienoic acid
HODTA: Hydroxyoctadecatetraenoic acid
HOTrE: Hydroxyoctadecatrienoic acid
HpETE: Hydroperoxyeicosatetraenoic acid
HPL: Hydroperoxide lyases
HpODE: Hydroperoxyoctadecadienoic acid
HpOTrE: Hydroperoxyoctadecatrienoic acid
IL: Interleukin
IMTA: Integrated multitrophic aquaculture
LOX: Lipoxygenases
MAPK: Mitogen-activated protein kinase
MDA: Malondialdehyde
NF-κB: Nuclear factor-κB
NO: Nitric oxide
PDA: Phytodienoic acid
PGHS: Prostaglandin endoperoxide H synthase
PKC: Protein kinase C
PLA_2_: Phospholipase A_2_
PPAR: Peroxisome proliferator-activated receptor
PUA: Polyunsaturated aldehydes
PUFA: Polyunsaturated fatty acids
RBL: Rat basophil leukemia
RES: Reactive electrophile species
ROS: Reactive oxygen species
Th: T helper
TNF: Tumor necrosis factor
UHPLC-QqQ-MS/MS: Ultrahigh-performance liquid chromatography coupled to triple-quadrupole mass spectrometry
